# Supplementary material for: ENSO phase transition enables prediction of winter North Atlantic Oscillation one year ahead
Source: Nat Commun. 2026 Mar 25;17:2588. doi: 10.1038/s41467-026-70646-2 (PMC13018643; doi:10.1038/s41467-026-70646-2)
Supplement: Supplementary file 1 — Supplementary Information [file 41467_2026_70646_MOESM1_ESM.pdf]

Supplementary Information

ENSO phase transition enables prediction of winter North Atlantic Oscillation one year ahead

Kiwook Kim[1], Myong-In Lee[1,*], Adam A. Scaife[2,3], and Doug M. Smith[2]

[1] Department of Civil, Urban, Earth and Environmental Engineering, Ulsan National Institute of Science and Technology, Ulsan, South Korea

[2] Met Office Hadley Centre, Exeter, United Kingdom

[3] Department of Mathematics and Statistics, University of Exeter, Exeter, United Kingdom

[*]Corresponding author.

E-mail: milee@unist.ac.kr

Fig. S1: NAO and ENSO prediction skill one year ahead in decadal prediction systems.
Fig. S2: The time series and prediction skill of DJF-mean NAO index.
Fig. S3: Changes in the North Atlantic variability depending on the phase characteristics of ENSO.
Fig. S4: Variations between NAO and SST depending on ENSO cycle.
Fig. S5: Lagged and concurrent dynamics between ENSO and NAO in the multi-model ensemble.
Fig. S6: Prediction skill of the ENSO index in all, ENSO transition, and persistence years.
Fig. S7: Probabilistic forecast of ENSO one year ahead.
Fig. S8: Seasonal evolution of NAO and ENSO variability during 1962–2019.
Fig. S9: Major interannual variation modes in the equatorial Pacific.
Fig. S10: Perfect-model method matched observational ENSO phase characteristics.
Table S1
Table S2
Table S3
Table S4

Supplementary Figures

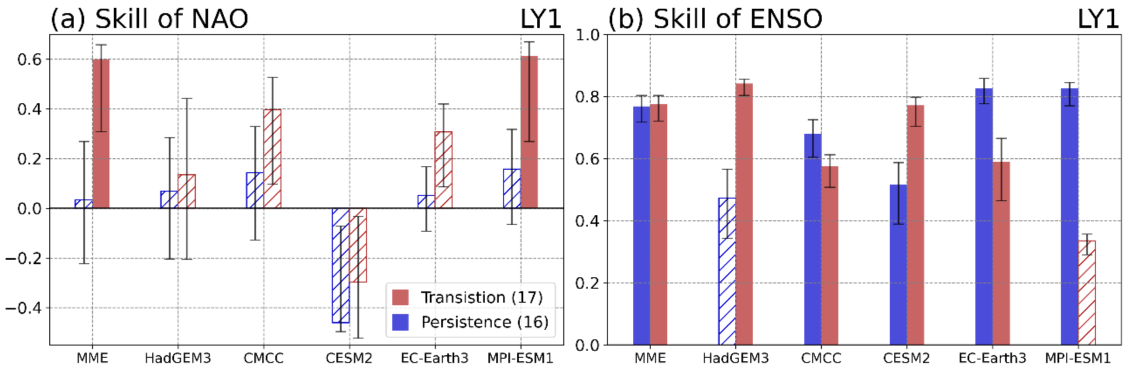

Fig. S1: NAO and ENSO prediction skill one year ahead in decadal prediction systems.

(a) Prediction skill of the JF(1)-mean NAO index at lead year 1 (LY1) during ENSO transition years (red, n = 17) and ENSO persistence years (blue, n = 16), for individual prediction systems and the multi-model ensemble (MME). **(b)** Prediction skill of the NDJ(1)-mean ENSO index by the MME and individual models initialized in November at LY0. Hatched bars indicate prediction skill that is not statistically significant at the 95% confidence level. Error bars denote the 10th–90th percentile range estimated from 1,000 bootstrap resamples for each system and period.

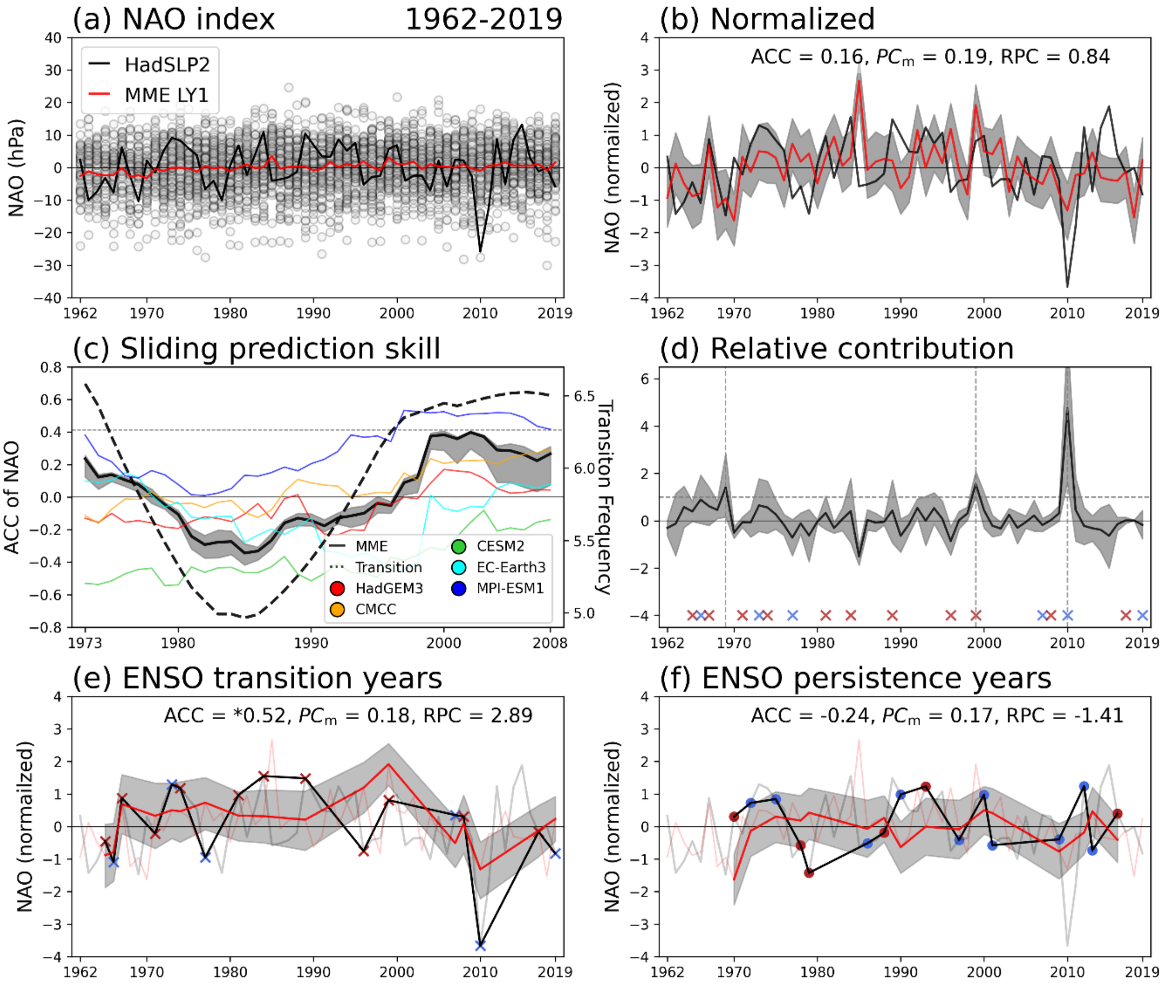

Fig. S2: The time series and prediction skill of DJF-mean NAO index.

Same as in **Fig. 1** except using the DJF-mean NAO index prediction.

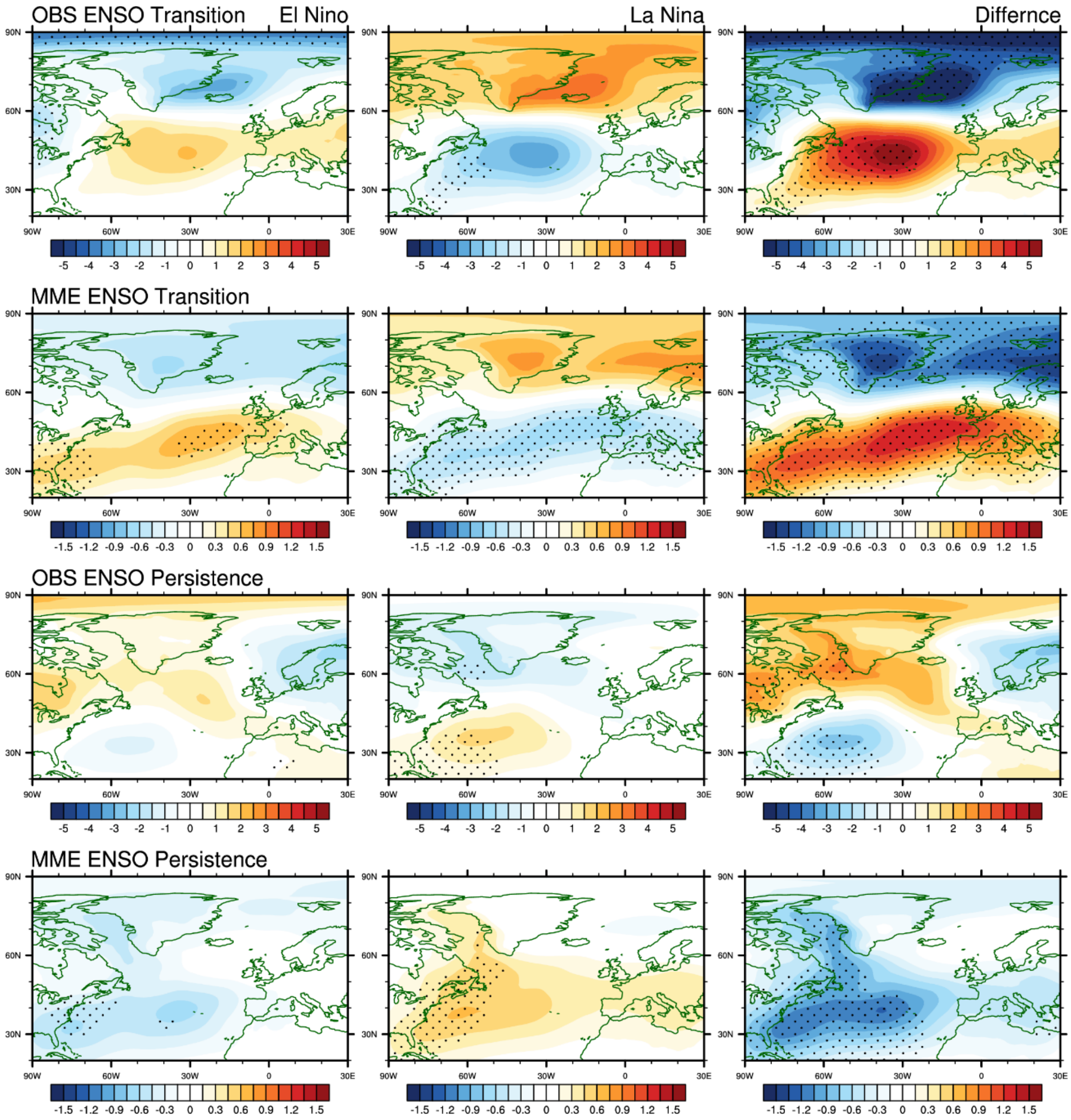

Fig. S3: Changes in the North Atlantic variability depending on the phase characteristics of ENSO.

Composite JF-mean SLP anomalies for El Niño and La Niña years. This figure includes and extends the results presented in **Fig. 2**, providing the full composite fields for ENSO-related SLP anomalies.

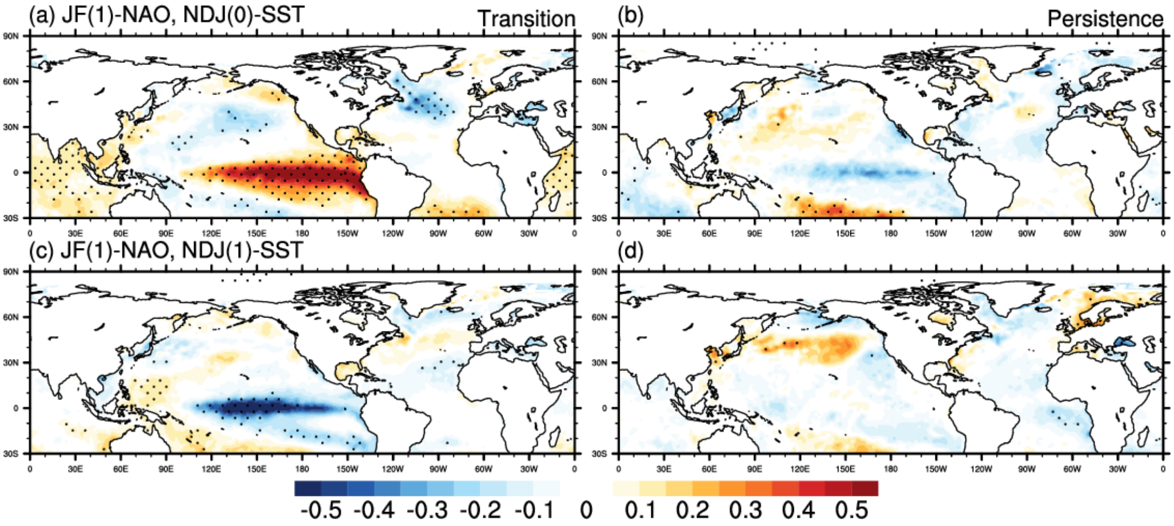

Fig. S4: Variations between NAO and SST depending on ENSO cycle.

Regression of observed NDJ(0)-mean SST anomalies (shaded, K) onto the JF(1)-mean NAO index for **(a)** ENSO transition years and **(b)** ENSO persistence years. **(c-d)** Same as **(a-b)**, but for NDJ(1)-mean SST anomalies. Stippling denotes statistical significance at the 95% confidence level.

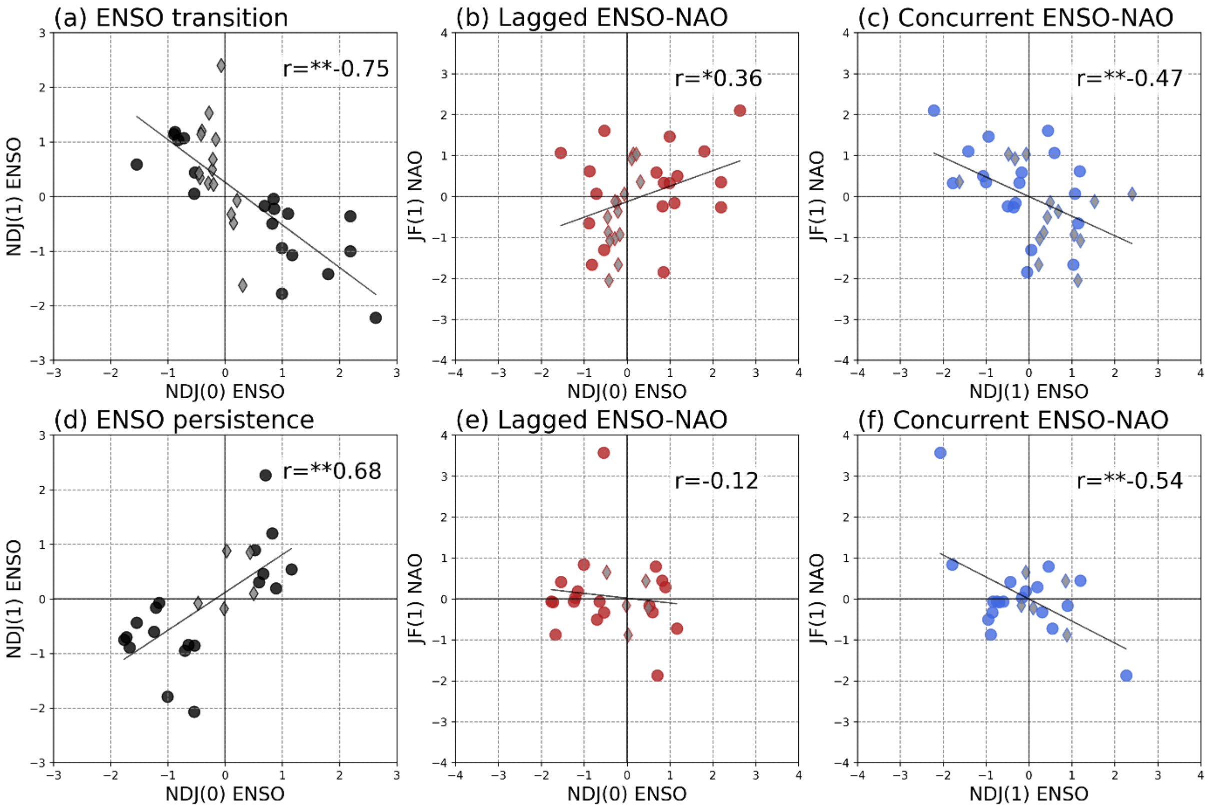

Fig. S5: Lagged and concurrent dynamics between ENSO and NAO in the multi-model ensemble.

Same as **Fig. 3**, but for the MME simulations, with statistical significance estimated from 1,000 bootstrap resamples.

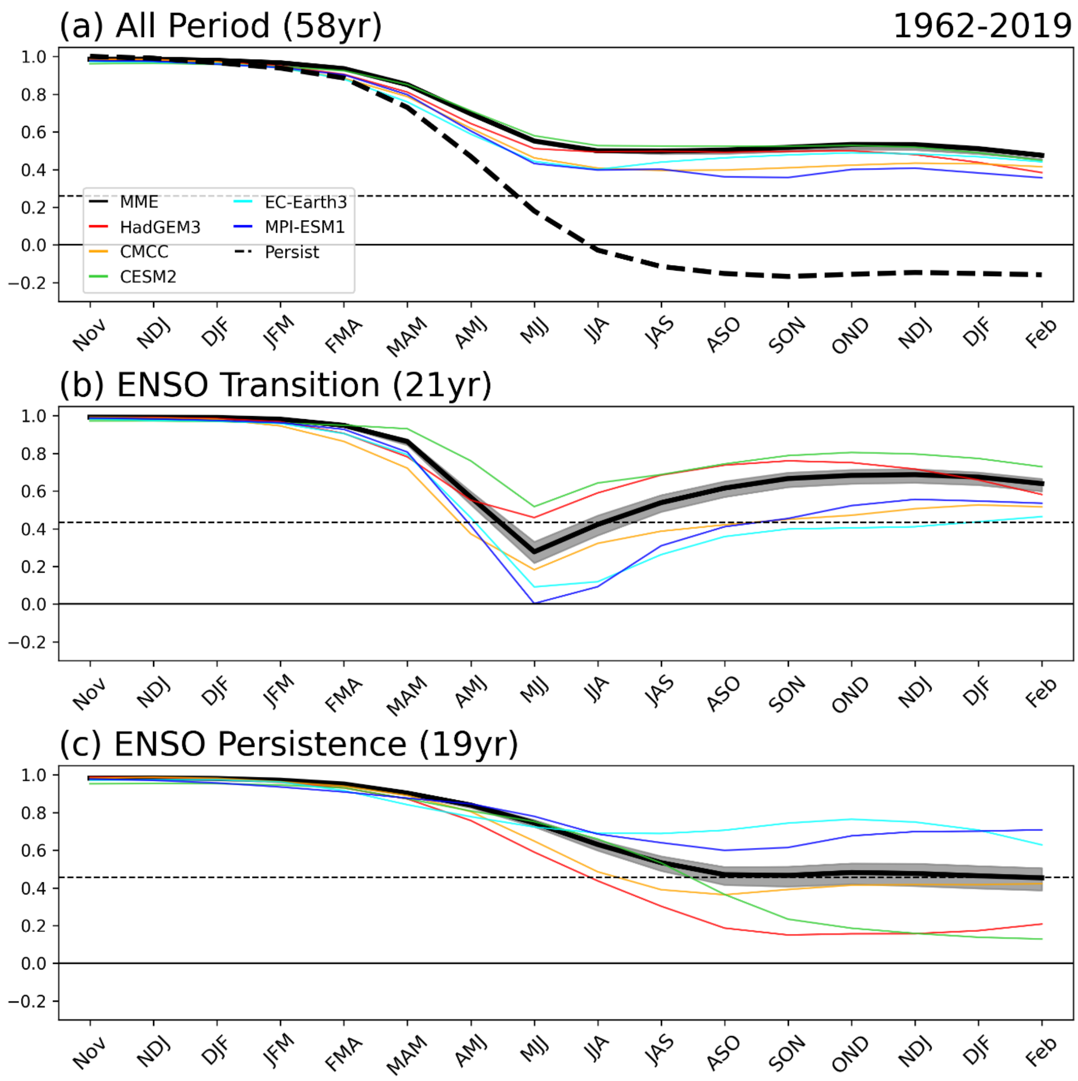

Fig. S6: Prediction skill of the ENSO index in all, ENSO transition, and persistence years.

(a) ACC of the 3-month-mean Niño3.4 index as a function of lead time (up to 15 months) for the MME (black) and individual decadal prediction systems (colored lines) during 1962-2019. The thick black dashed curve denotes the persistence forecast based on the November Niño3.4 index, and the horizontal dashed line indicates the 5% significance level. Shading around the MME shows the ensemble spread between the 10th and 90th percentiles estimated from 1,000 bootstrap resamples. **(b)** Same as **(a)**, but composited for ENSO transition years. **(c)** Same as **(a)**, but composited for ENSO persistence years.

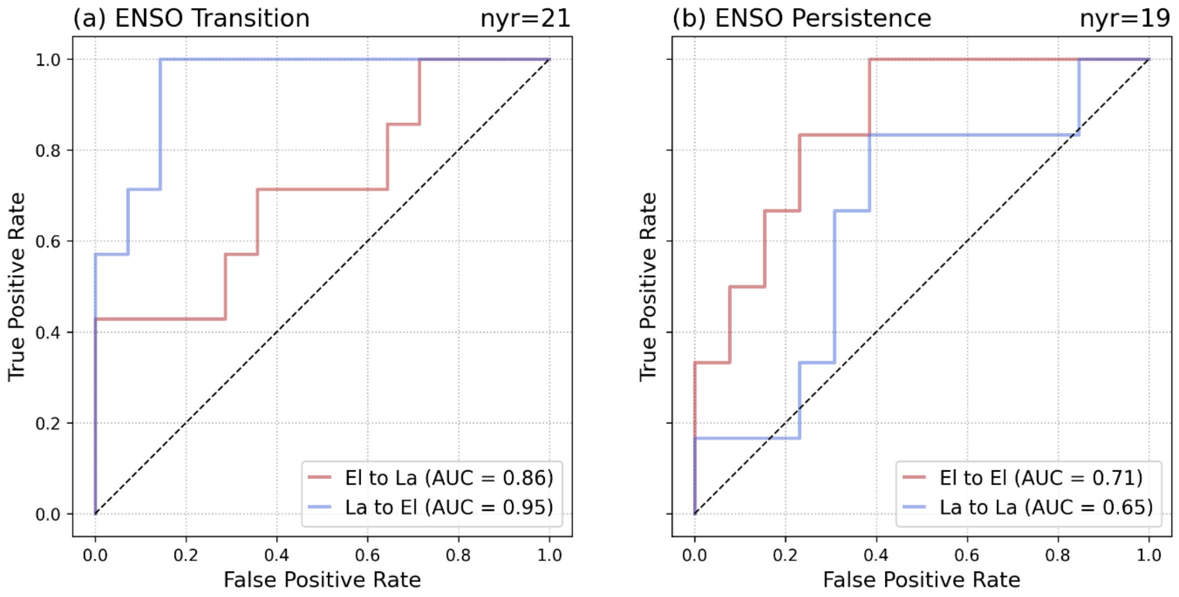

Fig. S7: Probabilistic forecast of ENSO one year ahead.

Receiver Operating Characteristic (ROC) curves for the NDJ(1) ENSO index predicted by the MME, evaluated for **(a)** ENSO transition years and **(b)** ENSO persistence years. The dashed diagonal line represents no-skill prediction. Area under the curve (AUC) values are shown in the legends, quantifying the forecast skill of the MME.

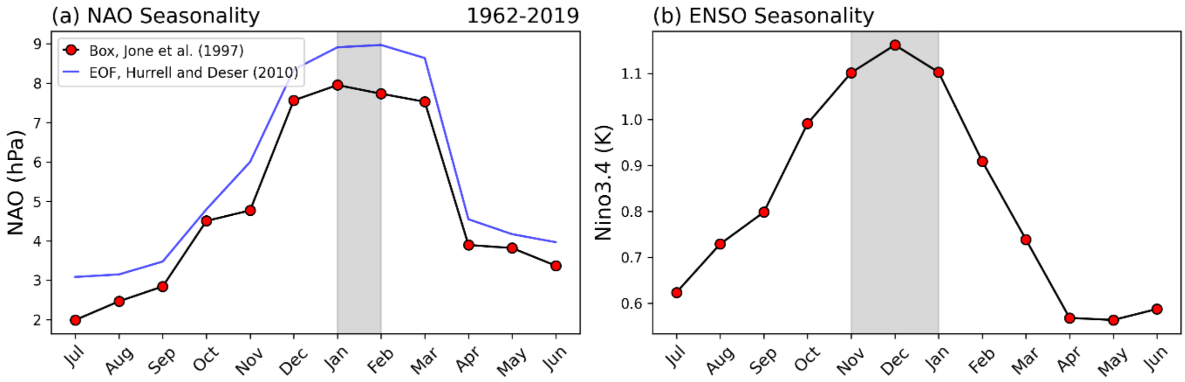

Fig. S8: Seasonal evolution of NAO and ENSO variability during 1962–2019.

(a) Monthly standard deviation of the NAO index. Red circles denote the NAO index following Jones et al. (1997)[1], while the blue line shows the EOF-based NAO index following Hurrell and Deser (2010)[2]. **(b)** Monthly standard deviation of the ENSO index. Gray shaded boxes indicate the target seasons during which variability is maximized and which are used for the main analysis: January–February (JF) for the NAO and November–December–January (NDJ) for ENSO.

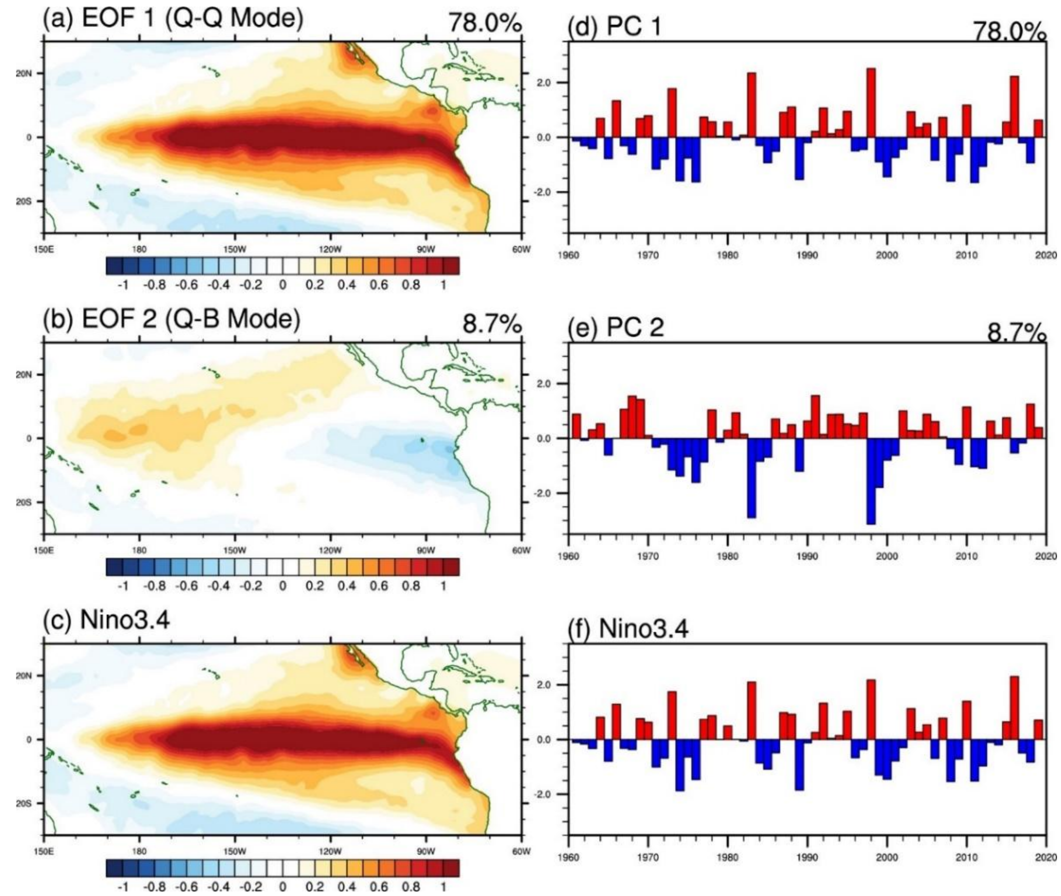

Fig. S9: Major interannual variation modes in the equatorial Pacific.

(a-b) Leading two EOF modes of observed NDJ-mean SST anomalies. **(c)** Spatial SST anomaly pattern associated with the Niño3.4 index. **(d-f)** Corresponding time series of PC 1, PC 2, and the Niño3.4 index.

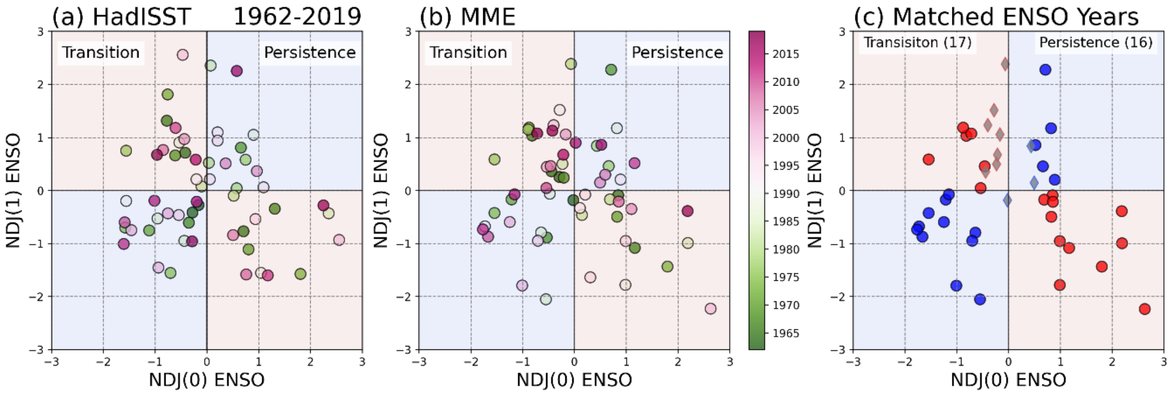

Fig. S10: Perfect-model method matched observational ENSO phase characteristics.

(a) Observed scatter plot of the NDJ(0)-mean ENSO index (x-axis) versus the NDJ(1)-mean ENSO index (y-axis) over 1962-2019. **(b)** Same as **(a)**, but for the ensemble-mean ENSO index predicted by the MME. Shading indicates the corresponding year. **(c)** Scatter plot of the MME for composite years in which the ENSO transition or persistence characteristics one year ahead are consistent between observations and the MME. Diamonds denote weak ENSO transition or persistence years at LY0. Red and blue boxes indicate ENSO transition and persistence categories, respectively. ENSO indices in the MME are estimated using 1,000 bootstrap resamples.

Supplementary Tables

Table S1: Observed NAO anomaly changes between the first winter JF(0) and the second winter JF(1), composited by ENSO evolution. ENSO transition and persistence are defined by the sign of the ENSO index in NDJ(1) relative to NDJ(0), using 0 K as the threshold. Each number shows the number of years, the average ΔNAO (JF(1) - JF(0), hPa), and the associated p-value. Bold values indicate statistical significance at the 5% level.

ENSO state in NDJ(1)	La Niña (NDJ(0))	Neutral (NDJ(0))	El Niño (NDJ(0))
positive (> 0 K)	**8, -1.02, 0.04**	12, -0.01, 0.94	7, 0.14, 0.74
negative (< 0 K)	12, 0.03, 0.82	6, -0.77, 0.40	**13, 0.74, 0.04**

Table S2: Summary of the decadal climate prediction systems used in this study.

Forecasting center	Forecasting System	Hindcast	Model (atm, ocn, land, ice, aero, chem)	Resolution	Ensemble cycle
Met Office, UK	HadGEM3-GC3.1[3]	1960-2017	HadGEM3-GA7.1, JULES-GL7.1, NEMO-GO6.0, CICE-GSI8, UKCA-GLOMAP	N216, L75	10 members on 1st day of November in each year
CMCC, Italy	CMCC-CM2-SR5[4]	1960-2017	CAM5.3, CLM4.5, NEMO3.6, CICE4.0, MAM3	0.9 x 1.25, L30	20 members on 1st day of November in each year
EC-Earth Consortium	EC-Earth3[5]	1960-2017	IFS cy36r4, HTESSEL, NEMO3.6, LIM3	T255, L91	10 members on 1st day of November in each year
MPI-M, Germany	MPI-ESM1-2-HR[6]	1960-2017	ECHAM6.3, JSBACH3.20, MPIOM1.63, HAMOCC6	T127, L95	10 members on 1st day of November in each year
NCAR, USA	CESM2-SMYLE[7]	1970-2017	CAM6.0, CLM5, POP2, CICE5, MARBL	0.9 x 1.25, L30	20 members on 1st day of Nov, Feb, May, Aug in each year

Table S3: Summary of the CMIP6 models used in this study.

Number	CMIP ID	Modeling Group	Reference
1	ACCESS-CM2	Commonwealth Scientific and Industrial Research Organization	Bi et al. (2013)[8]
2	ACCESS-ESM1-5		Law et al. (2017)[9]
3	AWI-CM-1-1-MR	Alfred Wegener Institute, Helmholtz Centre for Polar and Marine Research	Semmler et al. (2020)[10]
4	AWI-ESM-1-1-LR		Shi et al. (2020)[11]
5	BCC-CSM2-MR	Beijing Climate Center, China Meteorological Administration	Wu et al. (2019)[12]
6	BCC-ESM1		Wu et al. (2020)[13]
7	CanESM5	Canadian Centre for Climate Modelling and Analysis	Swart et al. (2019)[14]
8	CAMS-CSM1-0	Chinese Academy of Meteorological Sciences	Rong et al. (2019)[15]
9	CESM2	National Center for Atmospheric Research	Lauritzen et al. (2018)[16]
10	CESM2-FV2		Danabasoglu (2019a)[17]
11	CESM2-WACCM		Danabasoglu (2019b)[18]
12	CESM2-WACCM-FV2		Danabasoglu (2019c)[19]
13	CMCC-CM2-HR4	Fondazione Centro Euro-Mediterraneo sui Cambiamenti Climatici	Scoccimarro et al. (2020)[20]
14	CMCC-CM2-SR5		Cherchi et al. (2019)[21]
15	CMCC-ESM2		Lovato et al. (2022)[22]
16	FGOALS-g3	Chinese Academy of Sciences	Li et al. (2020)[23]
17	FIO-ESM-2-0	First Institute of Oceanography, Qingdao National Laboratory for Marine Science and Technology	Bao et al. (2020)[24]
18	GISS-E2-1-G	NASA Goddard Institute for Space Studies	Kelly et al. (2020)[25]

19	GISS-E2-1-G-CC		NASA/GISS (2019a)[26]
20	GISS-E2-1-H		NASA/GISS (2019b)[27]
21	HadGEM3-GC31-LL	Met Office Hadley Centre	Andrews et al. (2019)[28]
22	HadGEM3-GC31-MM		
23	MIROC6	Japan Agency for Marine-Earth Science and Technology, Atmosphere and Ocean Research Institute, National Institute for Environmental Studies and RIKEN Center for Computational Science	Tatebe et al. (2019)[29]
24	MIROC-ES2L		Hajima et al. (2020)[30]
25	MPI-ESM-1-2-HAM	HAMMOZ-Consortium	Neubauer et al. (2019)[31]
26	MPI-ESM1-2-HR	Max Planck Institute for Meteorology	Gutjahr et al. (2019)[6]
27	MPI-ESM1-2-LR		Mauritsen et al. (2019)[32]
28	MRI-ESM2-0	Meteorological Research Institute	Yukimoto et al. (2019)[33]
29	NESM3	Nanjing University of Information Science and Technology	Cao et al. (2018)[34]
30	SAM0-UNICON	Seoul National University	Park et al. (2019)[35]
31	TaiESM1	Research Center for Environmental Changes, Academia Sinica	Lee et al. (2020)[36]
32	UKESM1-0-LL	Met Office Hadley Centre	Sellar et al. (2019)[37]

Table S4: Classification of ENSO phase evolution and composite years used in this study. ENSO phase changes are defined based on transitions between NDJ(0) and NDJ(1). Numbers in parentheses indicate the total number of years in each category, and listed years denote the corresponding calendar years.

ENSO phase changes from NDJ(0) to NDJ(1)		
ENSO regime	ENSO evolution	Years
Transition (21)	El Niño → La Niña (9)	1965, 1971, 1974, 1989, 1996, 1999, 2006, 2008, 2011
	El Niño → weak La Niña (4)	1967, 1981, 1984, 2017
	La Niña → El Niño (8)	1966, 1969, 1973, 1977, 1987, 2007, 2010, 2019
	La Niña → weak El Niño (0)	N/A
Persistence (19)	El Niño → El Niño (4)	1970, 1978, 1988, 2016
	El Niño → weak El Niño (3)	1979, 1993, 2004
	La Niña → La Niña (8)	1972, 1975, 1976, 1986, 2000, 2001, 2009, 2012
	La Niña → weak La Niña (4)	1990, 1997, 2002, 2013

Supplementary References

1. Jones PD, Jonsson T, Wheeler D. Extension to the North Atlantic oscillation using early instrumental pressure observations from Gibraltar and south-west Iceland. *International Journal of Climatology* **17**, 1433–1450 (1997).

2. Hurrell JW, Deser C. North Atlantic climate variability: The role of the North Atlantic Oscillation. *Journal of Marine Systems* **79**, 231–244 (2010).

3. Williams KD*, et al.* The Met Office Global Coupled Model 3.0 and 3.1 (GC3.0 and GC3.1) Configurations. *Journal of Advances in Modeling Earth Systems* **10**, 357–380 (2018).

4. Nicolì D*, et al.* The Euro-Mediterranean Center on Climate Change (CMCC) decadal prediction system. *Geoscientific Model Development* **16**, 179–197 (2023).

5. Döscher R*, et al.* The EC-Earth3 Earth system model for the Coupled Model Intercomparison Project 6. *Geoscientific Model Development* **15**, 2973–3020 (2022).

6. Gutjahr O*, et al.* Max Planck Institute Earth System Model (MPI-ESM1.2) for the High-Resolution Model Intercomparison Project (HighResMIP). *Geoscientific Model Development* **12**, 3241–3281 (2019).

7. Yeager SG*, et al.* The Seasonal-to-Multiyear Large Ensemble (SMYLE) prediction system using the Community Earth System Model version 2. *Geoscientific Model Development* **15**, 6451–6493 (2022).

8. Bi D*, et al.* The ACCESS coupled model: description, control climate and evaluation. *Australian Meteorological and Oceanographic Journal* **63**, 41–64 (2013).

9. Law RM*, et al.* The carbon cycle in the Australian Community Climate and Earth System Simulator (ACCESS-ESM1) – Part 1: Model description and pre-industrial simulation.

Geoscientific Model Development **10**, 2567–2590 (2017).

10. Semmler T*, et al.* Simulations for CMIP6 With the AWI Climate Model AWI-CM-1-1. *Journal of Advances in Modeling Earth Systems* **12**, e2019MS002009 (2020).

11. Shi X, Yang H, Danek C, Lohmann G. AWI AWI-ESM1.1LR model output prepared for CMIP6 PMIP. (2020).

12. Wu T*, et al.* The Beijing Climate Center Climate System Model (BCC-CSM): the main progress from CMIP5 to CMIP6. *Geoscientific Model Development* **12**, 1573–1600 (2019).

13. Wu T*, et al.* Beijing Climate Center Earth System Model version 1 (BCC-ESM1): model description and evaluation of aerosol simulations. *Geoscientific Model Development* **13**, 977–1005 (2020).

14. Swart NC*, et al.* The Canadian Earth System Model version 5 (CanESM5.0.3). *Geoscientific Model Development* **12**, 4823–4873 (2019).

15. Rong X. CAMS CAMS-CSM1.0 model output prepared for CMIP6 ScenarioMIP. (2019).

16. Lauritzen PH*, et al.* NCAR Release of CAM-SE in CESM2.0: A Reformulation of the Spectral Element Dynamical Core in Dry-Mass Vertical Coordinates With Comprehensive Treatment of Condensates and Energy. *Journal of Advances in Modeling Earth Systems* **10**, 1537–1570 (2018).

17. Danabasoglu G. NCAR CESM2-FV2 model output prepared for CMIP6 CMIP historical. (2019).

18. Danabasoglu G. NCAR CESM2-WACCM model output prepared for CMIP6 CMIP. (2019).

19. Danabasoglu G. NCAR CESM2-WACCM-FV2 model output prepared for CMIP6 CMIP historical. (2019).

20. Scoccimarro E, Bellucci A, Peano D. CMCC CMCC-CM2-HR4 model output prepared for CMIP6 CMIP historical. (2020).

21. Cherchi A*, et al.* Global Mean Climate and Main Patterns of Variability in the CMCC-CM2 Coupled Model. *Journal of Advances in Modeling Earth Systems* **11**, 185–209 (2019).

22. Lovato T*, et al.* CMIP6 Simulations With the CMCC Earth System Model (CMCC-ESM2). *Journal of Advances in Modeling Earth Systems* **14**, e2021MS002814 (2022).

23. Li L*, et al.* The Flexible Global Ocean-Atmosphere-Land System Model Grid-Point Version 3 (FGOALS-g3): Description and Evaluation. *Journal of Advances in Modeling Earth Systems* **12**, e2019MS002012 (2020).

24. Bao Y, Song Z, Qiao F. FIO-ESM Version 2.0: Model Description and Evaluation. *Journal of Geophysical Research: Oceans* **125**, e2019JC016036 (2020).

25. Kelley M*, et al.* GISS-E2.1: Configurations and Climatology. *J Adv Model Earth Syst* **12**, e2019MS002025 (2020).

26. (NASA/GISS). NASA-GISS GISS-E2-1-G-CC model output prepared for CMIP6 CMIP. (2019).

27. (NASA/GISS). NASA-GISS GISS-E2.1H model output prepared for CMIP6 CMIP historical. (2019).

28. Andrews MB*, et al.* Historical Simulations With HadGEM3-GC3.1 for CMIP6. *Journal of Advances in Modeling Earth Systems* **12**, e2019MS001995 (2020).

29. Tatebe H*, et al.* Description and basic evaluation of simulated mean state, internal variability, and climate sensitivity in MIROC6. *Geoscientific Model Development* **12**, 2727–2765 (2019).

30. Hajima T*, et al.* Development of the MIROC-ES2L Earth system model and the evaluation of biogeochemical processes and feedbacks. *Geoscientific Model Development* **13**, 2197–2244

(2020).

31. Neubauer D*, et al.* HAMMOZ-Consortium MPI-ESM1.2-HAM model output prepared for CMIP6 CMIP historical. (2019).

32. Mauritsen T*, et al.* Developments in the MPI-M Earth System Model version 1.2 (MPI-ESM1.2) and Its Response to Increasing CO(2). *J Adv Model Earth Syst* **11**, 998–1038 (2019).

33. Yukimoto S*, et al.* The Meteorological Research Institute Earth System Model Version 2.0, MRI-ESM2.0: Description and Basic Evaluation of the Physical Component. *Journal of the Meteorological Society of Japan Ser II* **97**, 931–965 (2019).

34. Cao J*, et al.* The NUIST Earth System Model (NESM) version 3: description and preliminary evaluation. *Geoscientific Model Development* **11**, 2975–2993 (2018).

35. Park S, Shin J, Kim S, Oh E, Kim Y. Global Climate Simulated by the Seoul National University Atmosphere Model Version 0 with a Unified Convection Scheme (SAM0-UNICON). *Journal of Climate* **32**, 2917–2949 (2019).

36. Lee W-L*, et al.* Taiwan Earth System Model Version 1: description and evaluation of mean state. *Geoscientific Model Development* **13**, 3887–3904 (2020).

37. Sellar AA*, et al.* UKESM1: Description and Evaluation of the U.K. Earth System Model. *Journal of Advances in Modeling Earth Systems* **11**, 4513–4558 (2019).
